# Assessment of the Permeability of 3,4-Methylenedioxypyrovalerone (MDPV) across the Caco-2 Monolayer for Estimation of Intestinal Absorption and Enantioselectivity

**DOI:** 10.3390/ijms24032680

**Published:** 2023-01-31

**Authors:** Ana Sofia Almeida, Bárbara Silva, Fernando Remião, Carla Fernandes

**Affiliations:** 1 Laboratório de Química Orgânica e Farmacêutica, Departamento de Ciências Químicas, Faculdade de Farmácia, Universidade do Porto, Rua Jorge Viterbo Ferreira nº 228, 4050-313 Porto, Portugal; 2 Centro Interdisciplinar de Investigação Marinha e Ambiental (CIIMAR), Universidade do Porto, Terminal de Cruzeiros do Porto de Leixões, Avenida General Norton de Matos, s/n, 4450-208 Matosinhos, Portugal; 3 UCIBIO—Applied Molecular Biosciences Unit, REQUIMTE, Laboratory of Toxicology, Department of Biological Sciences, Faculty of Pharmacy, University of Porto, Rua de Jorge Viterbo Ferreira nº 228, 4050-313 Porto, Portugal; 4 Associate Laboratory i4HB—Institute for Health and Bioeconomy, Faculty of Pharmacy, University of Porto, Rua Jorge Viterbo Ferreira nº 228, 4050-313 Porto, Portugal

**Keywords:** Caco-2 cells, enantioselectivity, intestinal absorption, MDPV, permeability, synthetic cathinone heterocycle, UHPLC-UV

## Abstract

3,4-Methylenedioxypyrovalerone (MDPV) is a widely studied synthetic cathinone heterocycle mainly concerning its psychoactive effects. It is a chiral molecule and one of the most abused new psychoactive substances worldwide. Enantioselectivity studies for MDPV are still scarce and the extent to which it crosses the intestinal membrane is still unknown. Herein, an in vitro permeability study was performed to evaluate the passage of the enantiomers of MDPV across the Caco-2 monolayer. To detect and quantify MDPV, a UHPLC-UV method was developed and validated. Acceptable values within the recommended limits were obtained for all evaluated parameters (specificity, linearity, accuracy, limit of detection (LOD), limit of quantification (LOQ) and precision). The enantiomers of MDPV were found to be highly permeable across the Caco-2 monolayer, which can indicate a high intestinal permeability. Enantioselectivity was observed for the P_app_ values in the basolateral (BL) to apical (AP) direction. Furthermore, efflux ratios are indicative of efflux through a facilitated diffusion mechanism. To the best of our knowledge, determination of the permeability of MDPV across the intestinal epithelial cell monolayer is presented here for the first time.

## 1. Introduction

Synthetic cathinones are a vast group of chiral compounds derived from cathinone, an alkaloid found in Khat (Catha edulis) leaves [1]. Chewing fresh khat leaves, which contain many components such as alkaloids, flavonoids, amino acids, glycosides, sterols, vitamins, and minerals, has been a tradition for centuries in some cultures [2]. Derivatives of cathinone emerged in the 1930′s being synthesized, firstly, with a medicinal intent. Methcathinone was one of the first ever synthetic cathinones and was meant to reach the market as an antidepressant. Other examples are pyrovalerone, explored as a treatment for obesity, chronic fatigue, and lethargy, and methylone as a potential antidepressant and anti-Parkinson’s agent [3,4,5]. Nevertheless, these compounds never reached the market due to powerful addictive properties [6]. To this day, only the synthetic cathinone bupropion reached the market, being currently used as an antidepressant and a support to smoking cessation [7].

Nowadays, synthetic cathinones are one of the most reported groups of new psychoactive substances (NPS) with new derivatives emerging on the drug market every year with unknown properties [8,9]. Therefore, the development of studies with synthetic cathinones and their enantiomers is crucial to better understand their properties [10,11]. Although enantioselectivity studies are still scarce, differences between enantiomers have been found in several cases [10,12,13,14].

Drug substances can enter the body through several absorption sites, the gastrointestinal tract being the most important. The absorption through the gastrointestinal tract can be influenced by many factors such as the physicochemical properties of the drug, gastrointestinal motility, and food intake [15,16]. For chiral drugs, differential absorption may happen for the enantiomers, leading to different permeability. Enantioselectivity is not expected for passive diffusion, but it can occur when there is transport-mediated process involved [17]. To better understand drug absorption, permeability studies need to be performed.

**Figure 1:**
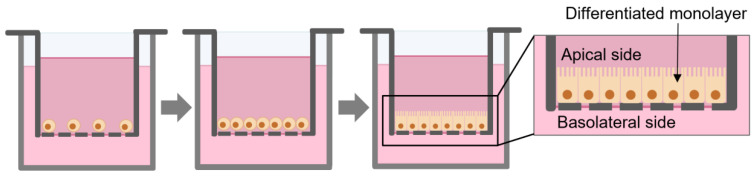
Scheme of the growth and differentiation of Caco-2 monolayer in permeable filters.

The Caco-2 cell line is one of the most used in vitro model for drug intestinal permeability and absorption studies [18]. Caco-2 cells are derived from human colorectal adenocarcinoma and can spontaneously differentiate into a polarized epithelial monolayer of cells (Figure 1) with tight junctions, microvilli, and several enzymes and transporters expressing most of morphological and functional properties of enterocytes. These characteristics provide this cell line the ability to mimic the small intestine [19,20].

Some studies have reported differences between the behavior of enantiomers of compounds in the permeability across the Caco-2 cell line [21,22]. For instance, when studying propranolol, a nonselective β-adrenoceptor blocker for the treatment of hypertension and cardiovascular disorders, *S*-propranolol was found to be the most transported in the apical (AP) to basolateral (BL) direction while *R*-propranolol was the most transported in the BL to AP direction [21].

After investigating the absorptive properties of Khat alkaloids in vitro, Atlabachew et al. [23] found that the transport across the mucosa of the oral cavity contributes significantly to the overall absorption of Khat alkaloids into the bloodstream and that they seem to be well absorbed into the gastrointestinal tract. Additionally, cathinone displayed significantly greater permeability than the other Khat alkaloids [23].

Although synthetic cathinones are widely studied, the extent to which these compounds cross the intestinal membrane is still unknown. In fact, the permeability across the gastrointestinal tract of synthetic cathinones has been only investigated for the enantiomers of pentedrone and methylone using the Caco-2 model [24]. Moreover, enantioselectivity was observed for both cathinones, *R*-(-)-pentedrone and *S*-(-)-methylone being the most permeable compounds [24].

**Figure 2:**
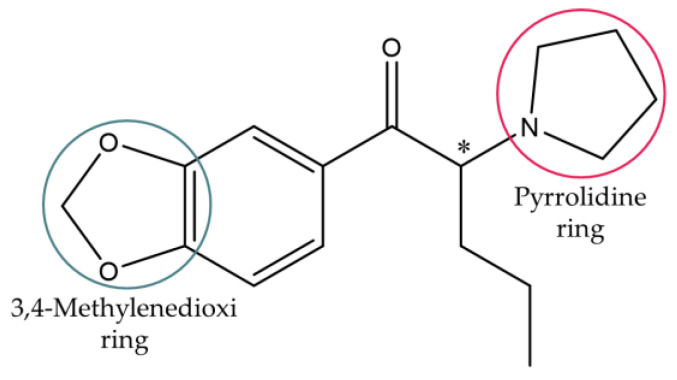
Chemical structure of MDPV.

This work focused on 3,4-methylenedioxypyrovalerone (MDPV), one of the most abused synthetic cathinones worldwide [25]. MDPV comprises a heterocyclic structure with a 3,4-methylenedioxi ring and a pyrrolidine ring (Figure 2), making this derivative part of the group of 3,4-methylenedioxypyrrolidinophenones or mixed cathinones [3].

The main goal of this work was to investigate the intestinal permeability across the gastrointestinal tract and potential enantioselectivity of MDPV using the in vitro Caco-2 model. To achieve that, the development and validation of an UHPLC-UV method for the detection and quantification of MDPV were performed. To the best of our knowledge, determination of the permeability of MDPV across the intestinal epithelial cell monolayer is presented here for the first time.

## 2. Results

### 2.1. Multi-Milligram Resolution of MDPV Enantiomers

MDPV enantiomers were obtained by a semi-preparative chiral liquid chromatography method based on a previous work [26]. Both enantiomers were collected with high enantiomeric purity, the enantiomeric ratios (e.r.) obtained were >99% for the first enantiomer, *S*-(-)-MDPV, and 95% for the second the enantiomer, *R*-(+)-MDPV.

### 2.2. Chromatographic Method

**Figure 3:**
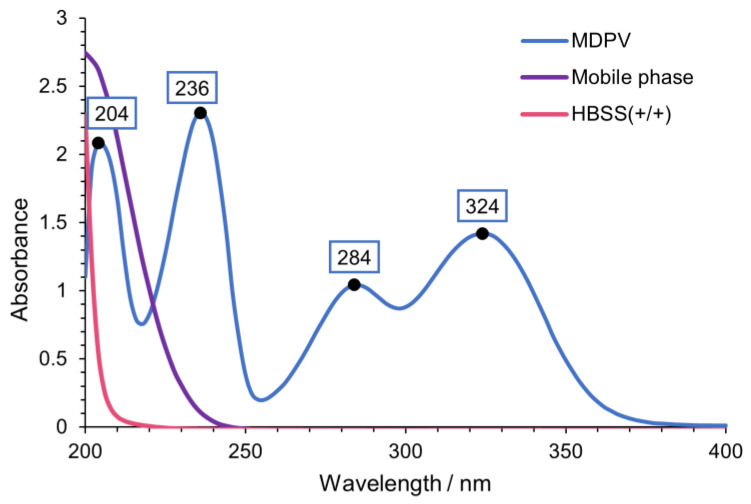
UV spectra of MDPV [100 µM in HBSS (+/+)], mobile phase [25 mM NH_4_CH_3_CO_2_: CH_3_CN: HCOOH (75:25:0.1 *v*/*v*/*v*)] and HBSS(+/+).

The chromatographic conditions of this work were based on Silva et al. [24] with some changes since a different cathinone and column were used. Flow rate was adjusted from 0.15 mL/min to 0.12 mL/min to allow a lower pressure and the mobile phase was adapted from 25 mM NH_4_CH_3_CO_2_:CH_3_CN:HCOOH (80:20:0.1 *v*/*v*/*v*) to 25 mM NH_4_CH_3_CO_2_: CH_3_CN: HCOOH (75:25:0.1 *v*/*v*/*v*). To obtain the optimal wavelength for the detection and quantification of MDPV, the ultraviolet (UV) spectrum of MDPV was determined (Figure 3). Four peaks with high absorption were found in the tested wavelength interval. Moreover, UV spectra were determined for the mobile phase and Hank’s balanced salt solution with calcium and magnesium [HBSS(+/+)], the buffer of the study, to detect potential interferences with MDPV absorption. The optimum wavelength selected for the detection and quantification of MDPV was 236 nm.

**Figure 4:**
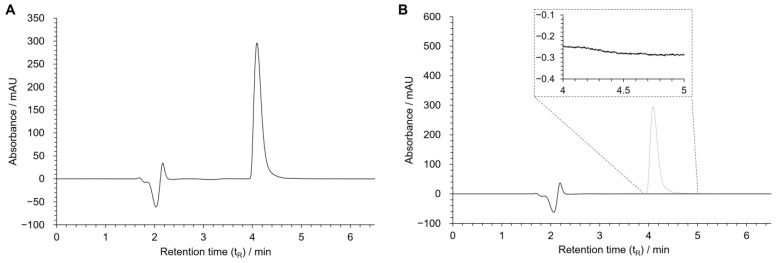
Chromatograms of MDPV [100 µM in HBSS(+/+)] (**A**), and blank sample [HBSS (+/+)] (**B**). Chromatographic conditions: Kinetex^®^ EVO C18 column, 25 mM NH_4_CH_3_CO_2_: CH_3_CN: HCOOH (75:25:0.1 *v*/*v*/*v*) as the mobile phase, flow rate: 0.12 mL/min, UV detection: 236 nm.

In the optimized chromatographic conditions, a good resolution was obtained for the peak of MDPV being eluted in less than 5 min (Figure 4A).

The samples from the permeability assay and calibration curves (all in HBSS (+/+)) were injected into the UHPLC after being filtered; no previous extraction or other treatment was needed.

### 2.3. Method Validation

#### 2.3.1. Specificity

To evaluate specificity, twenty blank samples containing only an HBSS (+/+) solution were injected and analyzed to detect potential chromatographic interferences with MDPV’s peak. No interference was observed between 4 and 5 min, the retention time corresponding to MDPV. The chromatogram of one of the blank samples is found in Figure 4B.

#### 2.3.2. Linearity

For the evaluation of linearity, five curves in a concentration range of 0.5–500 µM, independently prepared in five different days, were used to obtain the average linear regression equation and coefficient of determination (r^2^). An average r^2^ of 0.9999 was found. All linearity data are summarized in Table 1.

#### 2.3.3. Accuracy

After injecting three selected calibrators contained in the linear concentration interval (6 (low), 40 (medium), and 300 (high) μM) along with a calibration curve, the experimental concentrations of each calibrator were calculated through the linear regression equation of the curve and accuracy was measured through Equation (2). Percentages between 102% and 109% were obtained (Table 2).

#### 2.3.4. Precision

The results obtained for the evaluation of inter- and intra-day precisions of both equipment and method showed coefficients of variation (CV) between 2.88% and 13.87% (Table 3) [27,28].

#### 2.3.5. LOD and LOQ

Through Equations (3) and (4), using the slope of the average linear equation (Table 1) and the standard deviation of the analytical signal of 20 blank samples, a LOD of 0.063 µM and a LOQ of 0.190 µM were calculated for this method.

#### 2.3.6. Stability

Table 4 contains the peak area variation between the initial injection and final injection for each temperature tested, the obtained percentages being between 3.4% and 12.5%, which were, in general, low peak area variations. Moreover, peak areas were also compared to check for statistically significant differences (*p* < 0.05) between day 0 and each temperature and also between temperatures. No significant differences were observed.

### 2.4. Cell Viability Assay

**Figure 5:**
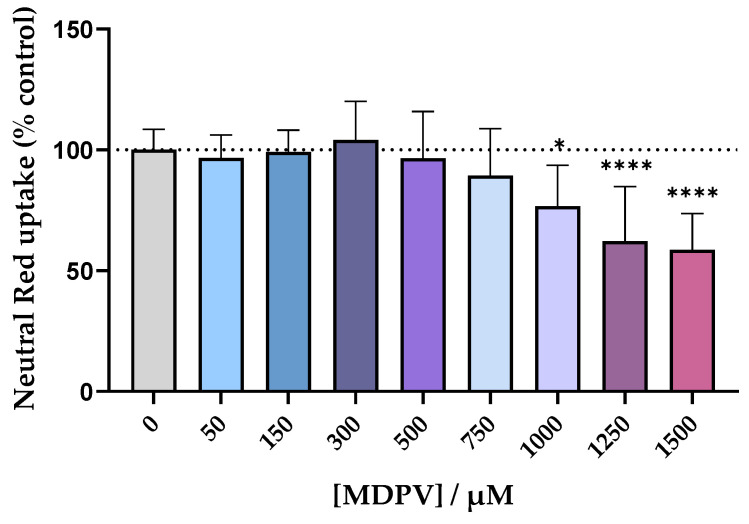
Cytotoxicity evaluation in Caco-2 cells exposed to racemic MDPV (0–1500 µM) for 24 h performed by the NR assay. Results are expressed as mean ± SD from four independent experiments (performed in triplicate). * *p* < 0.05, **** *p* < 0.0001 vs. control (0 µM).

In order to find a non-cytotoxic concentration to be used for the permeability assay with the Caco-2 cell line, the cells were exposed to racemic MDPV in a concentration range up to 1500 µM for 24 h and the Neutral Red (NR) assay was performed. The results, in Figure 5, showed no statistically significant differences in cell viability up to the concentration of 750 µM. The three highest concentrations tested resulted in a decrease of cell viability in a concentration dependent manner, so they were not considered for the assay. The concentration of 300 µM was selected for the permeability assay.

### 2.5. Permeability Assay

**Figure 6:**
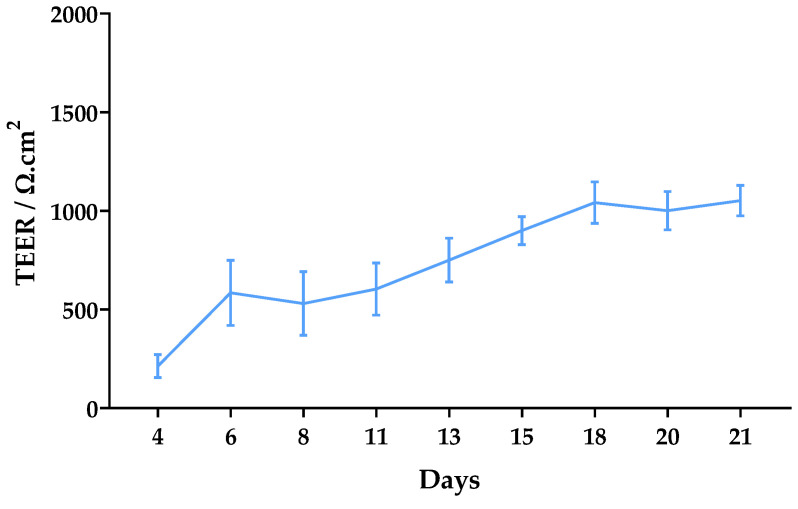
Monitoring of TEER values from day 4 to day 21 after seeding of Caco-2 cells. Results are expressed as mean ± SD obtained from three independent experiments.

The trans-epithelial electrical resistance (TEER) was monitored for 21 days after seeding (Figure 6). Significant TEER values were observed from day 6 (over 500 Ω·cm^2^). They gradually increased to approximately 1000 Ω·cm^2^ until day 18, after which they remained constant until day 21 with a final average value of 1052 Ω·cm^2^.

**Figure 7:**
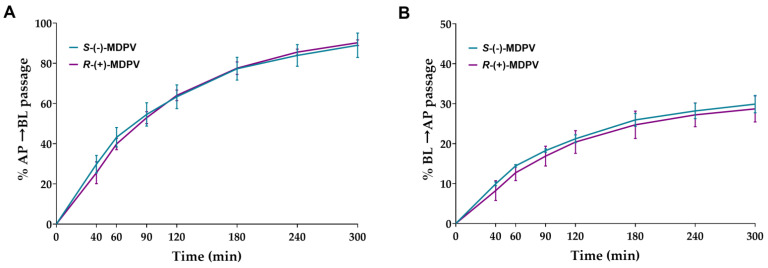
Percentage of cumulative quantity of the enantiomers of MDPV (*S*-(-)-MDPV and *R*-(+)-MDPV) transported through the Caco-2 monolayer in each time point for the AP to BL direction (**A**) and BL to AP direction (**B**). Results are expressed as mean ± SD obtained from three independent experiments (performed in duplicate).

Cells were exposed to 300 µM of each enantiomer on day 22 after seeding. Samples were collected in the chosen time points (40, 60, 90, 120, 180, 240, and 300 min) from the receiver compartment. After 6 weeks of storage, the validated UHPLC method was used to quantify MDPV present in the samples. Cumulative quantity transported from the donor compartment to the receiver compartment was calculated for each time point. The Results, shown in Figure 7, were expressed as the percentage of cumulative quantity in the initial quantity. A considerable percentage of passage was observed from the first time point (40 min) in both enantiomers for both directions, increasing significantly during the rest of the interval. No statistically significant difference was found between the enantiomers in any of the time points for both directions.

**Figure 8:**
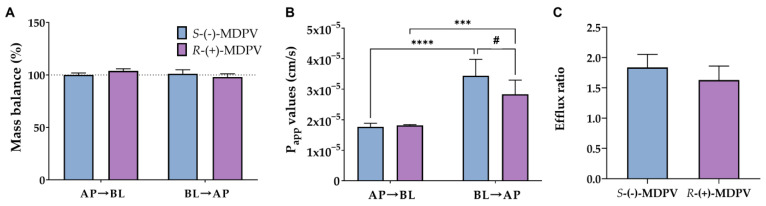
Mass balance (**A**), P_app_ values (**B**), and efflux ratios (**C**) obtained for the enantiomers of MDPV (*S*-(-)-MDPV and *R*-(+)-MDPV) after the permeability assay with Caco-2 cells. Results are expressed as mean ± SD obtained from three independent experiments (performed in duplicate). # *p* < 0.05 (between enantiomers), *** *p* < 0.001, **** *p* < 0.0001 (between directions).

Mass balance was calculated using Equation (5) (described in method section), the obtained values being between 98% and 104% (Figure 8A). Moreover, P_app_ values were calculated for the AP to BL direction using Equation (6) considering sink conditions (Figure 8B). Average P_app_ values of 1.8 × 10^−5^ and 1.81 × 10^−5^ cm/s were found for *S*-(-)-MDPV and *R*-(+)-MDPV, respectively, in this direction with no statistically significant differences between the enantiomers. For the BL to AP direction, Equation (7) was used to calculate P_app_ values under non-sink conditions (Figure 8B). Average P_app_ values of 3.4 × 10^−5^ and 2.8 × 10^−5^ cm/s were found for *S*-(-)-MDPV and *R*-(+)-MDPV, respectively, in this direction. In this case, statistically significant differences (*p* < 0.05) were found between the enantiomers. Additionally, when comparing the directions for each enantiomer separately, significant differences were found, the difference being more significant for *S*-(-)-MDPV (*p* < 0.001 for *R*-(+)-MDPV vs. *p* < 0.0001 for *S*-(-)-MDPV). Lastly, efflux ratios were calculated for each enantiomer using Equation (9) (Figure 8C). Efflux ratios of 1.8 for *S*-(-)-MDPV and 1.6 for *R*-(+)-MDPV were obtained with no statistically significant difference between enantiomers. 

The values obtained for mass balance, P_app_ values, and efflux ratios are summarized in Table 5.

## 3. Discussion

To evaluate the potential enantioselective intestinal absorption of MDPV, an in vitro permeability assay was performed using the Caco-2 cell line. The enantiomers of MDPV were separated by a semi-preparative chiral liquid chromatography method [26] with high enantiomeric purity. To analyze the passage of the MDPV enantiomers across the Caco-2 monolayer, a quantification method was needed. Here in, an UHPLC-UV method was selected based on previous work [24] and optimized for the conditions of this work. The optimized chromatographic conditions (Figure 4A) resulted in a well-resolved peak for MDPV and the analysis of the chromatograms of blank samples detected no interference between the buffer and MDPV (Figure 4B), which translated to a good specificity for the analysis and quantification of MDPV. All parameters were within recommended limits [27,28]. r^2^ values higher than 0.999 showed acceptable linearity in the analyzed concentration interval (0.5–500 μM) (Table 1). Likewise, acceptable accuracy was shown by percentages included in the recommended limits for this parameter (100 ± 15%) (Table 2) and for precision, CV values below 15% were observed (Table 3). LOQ and LOD values showed that the developed method displayed sensitivity for the detection and quantification of MDPV at a low micromolar concentration range. Thus, a UHPLC-UV method was successfully developed and validated for the detection and quantification of MDPV in HBSS (+/+).

Additionally, the stability of the samples was also evaluated after 6 weeks of storage at different temperatures. The calculated variation in peak area (Table 4) was in general low for every condition tested, meaning that the samples remained stable during the 6 weeks of storage.

The cytotoxic effects of racemic MDPV in Caco-2 cells were first assessed to find a non-cytotoxic concentration to further be used on the permeability assay. If Caco-2 cells were exposed to a cytotoxic concentration of MDPV, it could disrupt the cell monolayer, leading to erroneous results. On the other hand, if the concentration was too low, it could be harder to quantify. Thus, the concentration of 300 µM was selected (Figure 5).

To the best of our knowledge, apart from our previous work [24], no other studies with the Caco-2 cell line have been reported for synthetic cathinones. Thus, a close comparison was made with this study. For example, racemic pentedrone and methylone displayed no cytotoxic effects in Caco-2 cells in a concentration range up to 2000 μM [24]. Herein, a significant decrease in cell viability was observed starting at the concentration of MDPV of 1000 µM. Thus, MDPV seems to be more cytotoxic than both pentedrone and methylone in this cell line.

To better resemblance in vivo permeability conditions, the formation of a Caco-2 monolayer with good integrity is highly important. TEER values between 500 and 1100 Ω*cm^2^ are expected for fully differentiated monolayers [29]. In this work, values above 500 were observed from day 6 and, after 18 days of culture, values reached 1000 Ω*cm^2^ and remained constant until the assay (Figure 6). These results are indicative of good monolayer integrity.

No statistically significant differences were found in the transport of the enantiomers of MDPV through the Caco-2 monolayer in both directions in the time points selected (Figure 7). When comparing the results obtained for the enantiomers of pentedrone and methylone (only in the AP to BL direction) [24], the enantiomers of MDPV showed a higher extent of passage across the Caco-2 monolayer using a lower concentration. The presence of the pyrrolidine ring in the structure of MDPV, not present in both pentedrone and methylone, results in a decrease in the polarity of MDPV, which may lead to a greater diffusion of this cathinone derivate across cell membranes [30]. Thus, this structural difference could explain the greater passage of MDPV across the Caco-2 monolayer when compared with cathinones with the absence of that ring.

In this type of study, the calculation of the mass balance can be useful to understand if there were compound losses during the assay. A low mass balance can be caused by adsorption of the compound to the experimental material (plate or filter, for instance), metabolism or retention of the compound inside cells or cell membranes, which can, consequently, lead to errors in quantification of the compound and calculation of permeability coefficients [31]. In this work, values of mass balance were close to 100% (Figure 8A), suggesting that there were no compound losses during the assay.

Moreover, the calculation of P_app_ values provides an estimation of the permeability of compounds. When P_app_ values are higher than 1 × 10^−6^ cm/s, compounds are described as highly permeable substances while when P_app_ values are lower than 1 × 10^−6^ cm/s, compounds are considered weakly permeable substances [32]. Since P_app_ values were over 1 × 10^−6^ cm/s (Figure 8B), the enantiomers of MDPV were considered to be highly permeable across the Caco-2 monolayer, which can consequently suggest a high intestinal permeability. Additionally, significant differences (*p* < 0.05) were found between directions for both enantiomers, this difference being more significant for *S*-(-)-MDPV (*p* < 0.001 for *R*-(+)-MDPV vs. *p* < 0.0001 for *S*-(-)-MDPV). Although no significant differences were found in each time point between the enantiomers, significant differences (*p* < 0.05) were found in the P_app_ values for the BL to AP direction, suggesting enantioselectivity in the overall passage velocity of MDPV though the Caco-2 monolayer in that direction.

The efflux ratios were also calculated for each enantiomer. If higher than 2, this ratio is the first indicator of a potential involvement of an active transport process in the passage across the Caco-2 monolayer [31]. In this work, efflux ratios lower than 2 were found for both enantiomers with no statistically significant differences between them (Figure 8C). These results suggest that no active efflux should be excepted for the passage of MDPV across the Caco-2 monolayer. Nonetheless, P_app_ values for the BL to AP direction were significantly higher than the P_app_ values for the AP to BL direction and enantioselectivity was found between the enantiomers in P_app_ values in the BL to AP direction. Since enantioselectivity only occurs when a transport protein is involved [17], the involvement of a transport protein could be expected for the efflux of MDPV through facilitated diffusion, a passive-mediated transport that depends on a proton gradient [31]. For instance, some members of the solute carrier (SLC) family of transporters, such as OATP2B1 (involved in xenobiotics transport) or OCT1 (which transports protonated molecules), can be involved in facilitated diffusion and are expressed in Caco-2 cells [33,34,35].

## 4. Materials and Methods

### 4.1. Reagents

NH_4_CH_3_CO_2_ and CH_3_CN were acquired from Carlo Erba Reagents (Val de Reuil, FR). HCOOH, Dulbecco’s modified Eagle’s medium (DMEM) with 4.5 g/L glucose, Triton X-100, and the NR solution were purchased from Sigma-Aldrich (St. Louis, MO, USA). Antibiotic solution (10,000 U/mL penicillin, 10,000 μg/mL streptomycin), 0.25% trypsin/1 mM EDTA, Fetal bovine serum (FBS), and HBSS (+/+) were purchased from Gibco Laboratories (Lenexa, KS, USA).

### 4.2. Multi-Milligram Resolution of MDPV Enantiomers

Racemic MDPV (50:50 proportion of each enantiomer) was purchased from the Sensearomatics website (www.sensearomatics.eu, website unavailable currently). The MDPV enantiomers were obtained by a chiral liquid chromatography semi-preparative method [26] using a home-made column of tris-3,5-dimethylphenylcarbamate amylose coated onto aminopropylsilyl Nucleosyl (500 Å, 7 µm, 20%, *w*/*w*) and packed into a stainless-steel column (20 cm × 7.0 mm ID) [36].

Multiple injections of racemic MDPV were performed using Hexane:Ethanol:Diethylamine (97:3:0.1) as the mobile phase and a flow rate of 1.5 mL/min. Analyses were performed at 25 °C, in isocratic mode under UV detection (254 nm). Hydrochlorides were formed by precipitation of fractions of each enantiomer obtained with HCl on diethyl ether (2 M). Solutions for each enantiomer were prepared and reinjected to determine the e.r. obtained by the relative percentages of the peak areas [37]:

(1) $$e.r.\left(\%\right)=100\;\frac{\left[E_{1}\right]}{\left[E_{1}\right]+\left[E_{2}\right]}\;or\;100\;\frac{\left[E_{2}\right]}{\left[E_{1}\right]+\left[E_{2}\right]}$$

where [*S*-(-)-MDPV] and [*R*-(+)-MDPV] are the area of the peak of each enantiomer.

### 4.3. Instrumental and Chromatographic Conditions

A Thermo^®^ Scientific UHPLC with a Thermo^®^ Scientific Spectra System P4000 pump was used, with a Thermo^®^ Scientific Spectra AS3000 automatic injector and a Thermo^®^ Scientific Spectra System UV8000 model DAD. The software used to process the chromatographic data was ChromeleonTM 7.0. A Kinetex^®^ EVO C18 LC column (1.7 μm, 2.1 mm × 100 mm) connected to a SecurityGuard™ ULTRA pre-column (sub-2 µm, 2.1 mm × 2 mm). Chromatographic analyses were performed at room temperature at a flow rate of 0.12 mL/min using 25 mM NH_4_CH_3_CO_2_:CH_3_CN:HCOOH (75:25:0.1 *v*/*v*/*v*) as the mobile phase. The sample injection volume was 5 μL. Stock standards of 5 mM of MDPV and all solutions were prepared in HBSS (+/+) and filtered through a 13 mm nylon syringe filter with 0.22 µM pore size from Olimpeak™. Calibrators were prepared by the dilution of the stock standards with filtered HBSS (+/+) to final concentrations of 0.5, 1, 5, 10, 25, 50, 100, 250, and 500 μM. To obtain the optimal wavelength for the detection and quantification of MDPV, UV spectra were determined for MDPV (100 µM in HBSS (+/+)), the selected mobile phase and HBSS (+/+) using a UH5300 spectrophotometer.

### 4.4. Method Validation

The Food Drug Administration (FDA) and the International Conference on Harmonization (ICH) guidelines were followed to validate this method [27,28]. The parameters evaluated were specificity, linearity, accuracy, inter- and intraday precision, LOQ, and LOD. The stability of the analytes was also evaluated. Results were analyzed using the GraphPad Prism Software 9.0 and Microsoft Excel.

#### 4.4.1. Specificity

Twenty blank samples (HBSS (+/+)) were injected into the UHPLC to detect the presence of potential co-eluting peaks that could affect the analysis by the tested method.

#### 4.4.2. Linearity

To evaluate linearity, calibrators with concentrations ranging from 0.5 to 500 μM of MDPV (0.5, 1, 5, 10, 25, 50, 100, 250, and 500 μM) were independently prepared on five different days and injected into the UHPLC to obtain a calibration curve each day. The plot of peak area vs. concentration was analyzed by linear regression to obtain the r^2^.

#### 4.4.3. Accuracy

The accuracy of an analytical method represents the deviation between the experimental concentration and the expected (nominal) concentration as calculated by the following equation [27]:

(2) $$Accuracy\;\left(\%\right)=\frac{mean\;experimental\;concentration}{nominal\;concentration}\times 100$$

For the evaluation of this parameter, three different calibrators within the linear concentration range (6 (low), 40 (medium), and 300 (high) μM) were selected. Five independent solutions were prepared and injected in triplicate for each concentration. Furthermore, a calibration curve was injected and used to determine the experimental concentration of each calibrator.

#### 4.4.4. Precision

The precision of the methodology shows the proximity between a series of data acquired from several injections under the same conditions. The repeatability of the method can be determined for a short time interval (intra-day precision), or within different days (inter-day precision) [28]. Three calibrators, with concentrations within the linear concentration range (10 (low), 100 (medium) and 500 (high) μM), were selected for the evaluation of this parameter.

The inter-day precision of the equipment was evaluated by the preparation and injection in triplicate of the three calibrators in five consecutive days. For the determination of inter-day precision of the analytical method, the three calibrators were independently prepared and injected in triplicate on five consecutive days.

The intra-day precision of the equipment was evaluated by preparing and injecting five times the three calibrators on the same day. Lastly, for the determination of the intra-day precision of the analytical method, five solutions of the three calibrators were prepared independently and injected in triplicate on the same day.

Precision was expressed as the CV of each calibrator.

#### 4.4.5. LOD and LOQ

LOD and LOQ were calculated using the following equations [28]:

(3) $$LOD=\frac{3\sigma }{S}$$

(4) $$LOQ=\frac{10\sigma }{S}$$

where *σ* is the standard deviation of the analytical signal of 20 blank samples and *S* the slope of the calibration curve.

#### 4.4.6. Stability

To evaluate stability, three different calibrators within the linear concentration range (10 (low), 100 (medium), and 500 (high) μM) were prepared and injected into the UHPLC. The remaining volume of each calibrator was divided into several vials and further stored at different temperatures (room temperature, 4 °C, −20 °C, and −80 °C) for 6 weeks (the same storage time of the samples obtained from the permeability assay with Caco-2 cells). After the storage period, samples were injected in duplicate and the obtained analyte peak area for each calibrator in each temperature was compared with the peak area obtained on day 0. Percentages of peak variation were calculated for each concentration.

### 4.5. Caco-2 Cell Culture

The Caco-2 cell line was acquired from the European Collection of Cell Culture (ECACC, UK) and routinely maintained in DMEM supplemented with 10% FBS, 1% penicillin/streptomycin solution, and 1% non-essential amino acids at 37 °C in a humidified atmosphere of 5% CO_2_ with the medium changed every two days. Subcultures were obtained by trypsinization with a 0.25% trypsin/EDTA solution. For all assays, cells were used between the 14th and 20th passages.

### 4.6. Cell Viability Assay

MDPV cytotoxicity for the Caco-2 cell line was evaluated through the NR assay as previously described [38]. This method provides an indication of the ability of lysosomes from viable cells to incorporate the NR dye. Caco-2 cells were seeded onto 96-well plates using a density of 60,000 cells/cm^2^ to obtain confluent monolayers at the experimental day. The cells were incubated with the racemate of MDPV (0, 50, 150, 300, 500, 750, 1000, 1250, and 1500 µM) in fresh cell culture medium for 24 h. After the selected time interval, the cell culture medium was removed and replaced by a 50 µg/mL NR solution in HBSS (+/+) for 90 min, after which the NR solution was discarded, a lysis solution (50% EtOH/1% glacial acetic acid solution) was added, and the absorbance was read at 540 nm in a 96-well plate reader (PowerWaveX; Bio-Tek, Winooski, VT, USA). Additionally, 1% Triton X-100 was used as the positive control. Data were expressed as the percentage of cell viability relative to untreated cells. Data were obtained from four independent experiments performed in triplicate.

### 4.7. Permeability Assay

For the in vitro permeability assay, Caco-2 cells were seeded on polycarbonate transwell inserts (12-well, 0.4 μm pores, Corning) using a density of 120,000 cells/cm^2^ and cultivated for twenty-one days to allow the development of a differentiated monolayer. TEER was measured to control the integrity of the monolayers for twenty-one days.

After twenty-one days, the cells were incubated with 300 µM of each enantiomer of MDPV (in HBSS (+/+)). The assay was performed for both AP to BL and BL to AP directions. For AP to BL, exposure to the enantiomers was performed in the AP compartment (450 µL) while for BL to AP, exposure was made in the BL compartment (1250 µL). Right after the exposure (time 0), 50 µL was collected from each donor compartment (where exposure was performed). At suitable time intervals (40, 60, 90, 120, 180, 240, and 300 min), 600 µL (for AP to BL) and 200 µL (for BL to AL) of samples was collected from the BL and AP compartments (receiver compartments), respectively, and the same amount of HBSS (+/+) was added. In the last time point (300 min), 50 µL was also collected from the donor compartment. Collected samples were stored at −80 °C until the day of the UHPLC analysis. After 6 weeks of storage, samples were filtered with a 13 mm nylon syringe filter with 0.22 µM pore size from Olimpeak™ and quantification was performed by the validated UHPLC-UV method. Results were obtained for three experiments, performed in duplicate.

#### 4.7.1. Mass Balance

Mass balance was calculated using the following equation [31]:

(5) $$\%\;Mass\;balance=100\times \frac{Q_{R,T}+Q_{D,f}}{Q_{D,0}}$$

where *Q_R,T_* is the total cumulative quantity in the receiver chamber (nmol), *Q_D,f_* is the final quantity in the donor chamber (nmol), and *Q_D_*_,0_ is the initial quantity in the donor chamber (nmol).

#### 4.7.2. Permeability Coefficent (P_app_)

To calculate P_app_, first, a determination had to be made to determine whether the results were under sink or non-sink conditions. Sink conditions are considered if the ratio receiver concentration/donor concentration (C_R_/C_D_) at each sampling point is less than 10%. If the ratio is higher, P_app_ has to be calculated considering a non-sink analysis [39]. In this work, we found that the results from the AP to BL direction were under sink conditions while the results from the BL to AP direction were under non-sink conditions (Figure S1).

For sink conditions, P_app_ was calculated in cm/s using the following equation [24]:

(6) $$P_{app}=\frac{\Delta Q}{\Delta t}\times \frac{1}{A\times C_{0}}$$

where Δ*Q*/Δ*t* is the amount of compound over time (mol/s), *A* is the surface area of the monolayer (cm^2^), and *C*_0_ is the initial drug concentration on the donor side (mol/mL).

For non-sink conditions, a continuous change of the donor and receiver concentrations is considered, and the following equation must be used for each time interval to calculate the theoretical concentration at the receiver side:

(7) $$C_{R\left(t\right)}=\left(\frac{Q_{tot}}{V_{D}+V_{R}}\right)+\left(C_{R\left(t-1\right)}\times f-\frac{Q_{tot}}{V_{D}+V_{R}}\right)\times e^{-P\times A\times \left(\frac{1}{V_{R}}+\frac{1}{V_{D}}\right)\times \Delta t}$$

where *C_R_*_(*t*)_ is the theoretical concentration in the receiver side at time *t* (µM), *Q_tot_* is the total amount of drug in both chambers at time *t* (nmol), *V_R_* and *V_D_* are the volumes in the receiver and donor compartments (mL), respectively, *C_R_*_(*t*-1)_ is the concentration in the receiver chamber at the previous time (µM), *f* is the dilution factor for the sample replacement, *A* is the surface area of the monolayer (cm^2^), Δ*t* is the time interval (s), and *P* is an initial approximation of the permeability coefficient calculated through Equation (6) (cm/s).

P_app_ values are determined by the minimization of the Sum of Squared Residuals (SSR):

(8) $$SSR=\sum {\left[C_{R\left(t\right)theor}-C_{R\left(t\right)exp}\right]}^{2}$$

where *C_R_*_(*t*)*theor*_ is the theoretical concentration in the receiver side at time *t* calculated through Equation (7) and *C_R_*_(*t*)*exp*_ is the experimental concentration in the receiver side at time *t* obtained directly from the quantification of the samples.

A more in-depth explanation of sink/non-sink conditions and the application of these equations can be found in Tavelin et al. [39].

#### 4.7.3. Efflux Ratio

The efflux ratio, ratio between the permeability coefficients obtained for each direction, was calculated using the following equation [31]:

(9) $$Efflux\;ratio=\frac{P_{app\;}\left(BL\to AP\right)}{P_{app\;}\left(AP\to BL\right)}$$

### 4.8. Statistical Analysis

All statistical calculations were performed using GraphPad Prism 9.0 for Windows (GraphPad Software, San Diego, CA, USA) and Microsoft Excel. Kolmogorov–Smirnov and Shapiro–Wilk normality tests were used to evaluate the normality of the data distribution. For the cytotoxicity studies, the statistical comparisons were performed using one-way ANOVA, followed by Holm–Sidak’s multiple comparisons test. For the permeability experiment, one/two-way ANOVA was used to make statistical comparisons, followed by Holm–Sidak’s/Tukey’s multiple comparisons test. Differences were considered significant for *p* values lower than 0.05.

## 5. Conclusions

An UHPLC-UV method was successfully validated for the detection and quantification of MDPV in the transport buffer of this study, HBSS (+/+). The results showed that the enantiomers of MDPV were highly permeable across the Caco-2 monolayer. Enantioselectivity was found between the enantiomers in the P_app_ values obtained for the BL to AP direction, suggesting the involvement of a transport protein. Efflux ratios indicate that a facilitated diffusion mechanism should be excepted for the efflux of the enantiomers of MDPV. To the best of our knowledge, this is the first study related to the permeability of MDPV enantiomers across the intestinal epithelial cell monolayer. More studies with other synthetic cathinones and their enantiomers should be performed.

## Figures and Tables

**Table 1:** Linear regression data for evaluation of linearity.

Linear Equation	r^2^	95% Confidence Intervals		
		Slope	Y-Intercept	X-Intercept
0.5524x + 0.4222	0.9999	0.5486 to 0.5562	−0.2965 to 1.1409	−2.073 to 0.5348

**Table 2:** Accuracy data.

Nominal Concentration (µM)	Experimental Concentration (µM)	Accuracy (%)
6	6.1 ± 0.6	102 ± 10
40	44 ± 2	109 ± 6
300	315 ± 22	105 ± 7

**Table 3:** Inter-day and intra-day precision data.

Concentration (µM)	Inter-Day (CV %)		Intra-Day (CV %)	
	Equipment	Method	Equipment	Method
10	6.04	13.87	4.75	10.18
100	9.10	4.74	3.96	10.56
500	9.19	2.88	3.21	8.27

**Table 4:** Sample stability after 6 weeks of storage expressed as the % peak area variation relative to day 0.

Concentration (µM)	Storage Temperature			
	RT	4 °C	−20 °C	−80 °C
10	11.0	10.9	12.5	7.1
100	11.9	9.1	10.4	8.6
500	10.2	3.4	5.3	6.8

**Table 5:** Data obtained for mass balance, P_app_ values, and efflux ratios expressed as mean ± SD. # *p* < 0.05 (between enantiomers), *** *p* < 0.001, **** *p* < 0.0001 (between directions).

		Mass Balance (%)	P_app_	Efflux Ratio
*S*-(-)-MDPV	AP → BL	100 ± 2	1.8 × 10^−5^ ± 1 × 10^−6^ ****	1.8 ± 0.2
	BL → AP	101 ± 4	3.4 × 10^−5^ ± 1 × 10^−6^ ****^; #^	
*R*-(+)-MDPV	AP → BL	104 ± 2	1.81 × 10^−5^ ± 3 × 10^−7^ ***	1.6 ± 0.2
	BL → AP	98 ± 3	2.8 × 10^−5^ ± 5 × 10^−6^ ***^; #^	

## Data Availability

Data is contained within the article or supplementary material.

## Supplementary Materials

The following supporting information can be downloaded at: https://www.mdpi.com/article/10.3390/ijms24032680/s1.
