# Supplementary material for: Assessment of the Permeability of 3,4-Methylenedioxypyrovalerone (MDPV) across the Caco-2 Monolayer for Estimation of Intestinal Absorption and Enantioselectivity
Source: Int J Mol Sci. 2023 Jan 31;24(3):2680. doi: 10.3390/ijms24032680 (PMC9917214; doi:10.3390/ijms24032680)
Supplement: Supplementary file 1 [file ijms-24-02680-s001.zip › ijms-2139943-supplementary.pdf]

Supplementary Material

Assessment of the Permeability of 3,4-Methylenedioxypyrovalerone (MDPV) across the Caco-2 Monolayer for Estimation of Intestinal Absorption and Enantioselectivity

Ana Sofia Almeida [1,2,3,4], Bárbara Silva [3,4], Fernando Remião [3,4,*] and Carla Fernandes [1,2,*]

1 Laboratório de Química Orgânica e Farmacêutica, Departamento de Ciências Químicas, Faculdade de Farmácia, Universidade do Porto, Rua Jorge Viterbo Ferreira nº 228, 4050-313 Porto, Portugal
2 Centro Interdisciplinar de Investigação Marinha e Ambiental (CIIMAR), Universidade do Porto, Terminal de Cruzeiros do Porto de Leixões, Avenida General Norton de Matos, s/n, 4450-208 Matosinhos, Portugal
3 UCIBIO—Applied Molecular Biosciences Unit, REQUIMTE, Laboratory of Toxicology, Department of Biological Sciences, Faculty of Pharmacy, University of Porto, Rua de Jorge Viterbo Ferreira nº 228, 4050-313 Porto, Portugal
4 Associate Laboratory i4HB—Institute for Health and Bioeconomy, Faculty of Pharmacy, University of Porto, Rua Jorge Viterbo Ferreira nº 228, 4050-313 Porto, Portugal
* Correspondence: cfernandes@ff.up.pt (C.F.); remiao@ff.up.pt (F.R.)

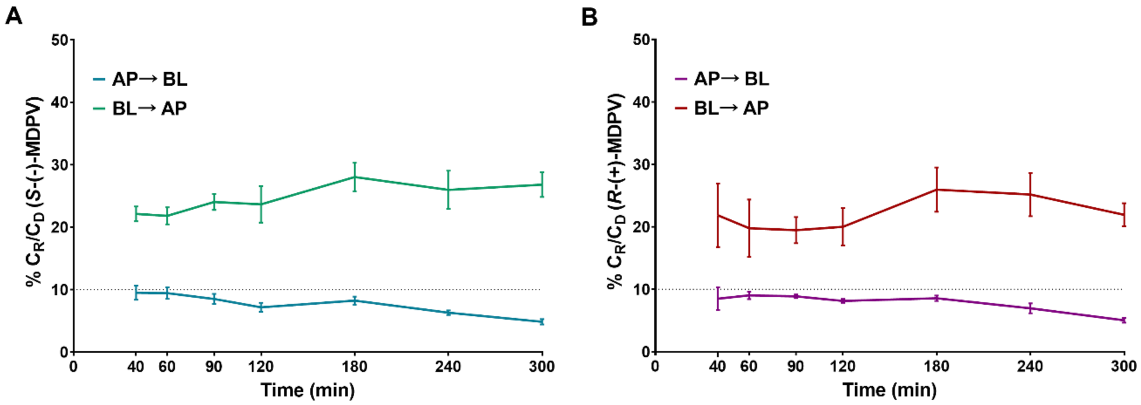

Figure S1. Ratio of C_R/C_D at each time point of sample collection (**A** – *S*-(-)-MDPV and **B** – *R*-(+)-MDPV). If lower than 10%, sink conditions are considered. If higher than 10%, non-sink conditions are considered.
